# Evaluation of prevalance and risk factors for bloodstream infection in severe coronavirus disease 2019 (COVID-19) patients

**DOI:** 10.1017/ash.2021.254

**Published:** 2022-02-21

**Authors:** Kubra Erbay, Hasan Selcuk Ozger, Ozlem Guzel Tunccan, Ümmügülsüm Gaygısız, Merve Buyukkoruk, Fidan Sultanova, Mehmet Yıldız, Nazlıhan Boyacı Dündar, Müge Aydoğdu, Gulendam Bozdayi, Murat Dizbay

**Affiliations:** 1 Department of Infectious Diseases and Clinical Microbiology, Gazi University School of Medicine, Ankara, Turkey; 2 Division of Critical Care Medicine, Department of Anesthesiology and Reanimation, Gazi University School of Medicine, Ankara, Turkey; 3 Division of Critical Care Medicine, Department of Internal Medicine, Gazi University School of Medicine, Ankara, Turkey; 4 Department of Pulmonary Critical Care Medicine, Gazi University School of Medicine, Ankara, Turkey; 5 Department of Medical Microbiology, Gazi University School of Medicine, Ankara, Turkey

**Keywords:** Bacteremia, Bloodstream infection, COVID-19, Intensive care unit

## Abstract

**Objectives:**

In this study, we sought to determine the prevalence of bloodstream infection (BSI) in severe coronavirus disease 2019 (COVID-19) patients and to determine the risk factors of BSI in critical COVID-19 patients.

**Design:**

Retrospective, descriptive study between March 2020 and January 2021.

**Setting:**

An 1,007-bed university hospital.

**Participants:**

Patients who were hospitalized due to severe COVID-19 disease and had an aerobic blood culture taken at least once during hospitalization

**Methods:**

Case definitions were made according to National Institutes of Health clinical definitions. According to the blood culture results, the patients were grouped as with and without BSIs, and compared for BSIs risk factors.

**Results:**

In total, 195 patients were included in the study. Blood culture positivity was detected in 76 (39.0%) of 196 patients. Excluding blood culture positivity considered as contamination, the prevalence of BSI in all severe COVID-19 cases was 18.5% (n = 36). In intensive care unit patients the prevalence of BSI was 30.6% (n = 26). In multivariate analyses, central venous catheter (odds ratio [OR], 8.17; 95% confidence interval [CI], 2.46–27.1; *P* < .01) and hospitalization in the multibed intensive care unit (OR, 4.28; 95% CI, 1.28–14.3; *P* < .01) were risk factors associated with the acquisition of BSI.

**Conclusion:**

The prevalence of BSI in COVID-19 patients is particularly high in critically ill patients. The central venous catheter and multibed intensive care follow-up are risk factors for BSI. BSIs can be reduced by increasing compliance to infection control measures and central venous catheter insertion-care procedures. The use of single-bed intensive care units where compliance can be achieved more effectively is important for the prevention of BSIs.

The coronavirus disease 2019 (COVID-19) pandemic, which emerged in Wuhan in December 2019 and spread rapidly all over the world, reached 212 million cases worldwide as of August 23, 2021.^1^ Moreover, 14% of cases have required hospitalization with severe pneumonia and 5% require intensive care support.^2^ Coinfection develops in 3.5% of COVID-19 patients and secondary bacterial infection develops in 14.3%.^3^ Among these infections, the most common infections after pneumonia are bloodstream infections (BSIs).^4–6^

The frequency of BSI reported in COVID-19 patients varies widely, ranging from 1.6% to 40%, depending on disease severity and infection definitions.^7–10^ Most of these infections have been identified in intensive care unit (ICU) patients because of the high risk of nosocomial secondary infections, associated with the severity of baseline conditions (APACHE II scores at ICU admission, oxygen support requirements at hospital admissions etc.), comorbidities (diabetes mellitus etc.), treatments and invasive procedures (intubations, central venous catheter (CVC) etc.).^6,11^ Detecting risk factors for BSI in these heterogeneous ICU patients is quite difficult. We sought to determine the prevalence of BSI in severe COVID-19 patients and to determine the risk factors of BSI in critical COVID-19 patients.

## Method

### Study design

This study was conducted between March 2020 and January 2021 as a single-center, retrospective, descriptive study. The study was approved by the Gazi University Clinical Research Ethics Committee (approval date March 29, 2021; approval no. 326).

### Study group and definitions

We included the following patients in this study: patients who were hospitalized due to severe COVID-19 disease with positive polymerase change reaction assay for severe acute respiratory coronavirus virus 2 (SARS-CoV-2) positivity in nasopharyngeal swab samples, who were aged ≥18 years, and who had an aerobic blood culture taken at least once during hospitalization.

Case definitions were made according to National Institutes of Health clinical definitions^12^: (1) Severe COVID-19 patient had oxygen saturation (SpO_2_) <94% on room air at sea level, a ratio of arterial partial pressure of oxygen to fraction of inspired oxygen (PaO2/FiO–) <300 mm Hg, respiratory frequency >30 breaths per minute, or lung infiltrates >50%. And (2) critical COVID-19 patient who had respiratory failure, septic shock, and/or multiple organ dysfunction.

According to the blood culture results, the patients were grouped as developing and not developing BSIs. If the number of developing BSIs was ≥2, the first BSI was included. BSI was defined using the Centers for Disease Control and Prevention (CDC) criteria as follows^13^: (1) Isolation of at least 1 of the microorganisms (*Staphylococcus aureus*, *Enterococcus* spp, and gram-negative bacteria) considered as BSI agents in the blood cultures taken; (2) microorganisms (coagulase-negative staphylococci, *Corynebacterium* spp, and *Bacillus* spp) associated with contamination and isolated in at least 2 blood cultures taken at different times; and (3) least 1 of the clinical manifestations of BSI (ie, fever ≥38.0°C, chills, or hypotension).

COVID-19 patients were followed in 2 different types of ICUs: (1) multibed ICU with a distance of 1 m between patient beds but where patients are in the common area (without negative pressure) and personal protective equipment is used to enter the intensive care area and (2) single-room ICU where the patients are in single, negative-pressure rooms with separate personal protective equipment at the entrance of each patient room.

### Study protocol

Patients who met the inclusion criteria defined above were included in the study. The following patient characteristics were recorded and evaluated: age, sex, comorbidities, clinical findings (symptoms at the time of admission to the hospital, symptom duration, vital signs at the time of hospitalization), laboratory results at hospital admission, antimicrobial and antiinflammatory treatments applied during hospitalization. The blood culture results of the patients during their hospitalizations were evaluated using the following information: hospital information system, the number of blood cultures taken, the time of blood culture, and microorganisms grown.

Clinical findings of critical COVID-19 patients at the time of ICU admission and during intensive care follow-up (ie, fever, Acute Physiology and Chronic Health Evaluation (APACHE-2) score, Sequential Organ Failure Assessment (SOFA) score, vital signs, type of respiratory support, requirement of vasoactive agents), total parenteral nutrition, central venous catheter (CVC), renal replacement therapy, laboratory results at the time of admission to the ICU and at the time of blood culture positivity, and antimicrobial and anti-inflammatory treatments applied during the ICU stay were evaluated and recorded. Acute renal failure evaluated during blood culture positivity was defined according to the Kidney Disease, Improving Global Outcomes (KDIGO) criteria.^14^ Nosocomial infection incidence density, hand hygiene compliance rates, patient-healthcareprofessional ratios in COVID-19 ICU were evaluated and compared with non-COVID-19 ICU according to 2020-2021 hospital surveillance data.

### Statistical analyses

Statistical Package for the Social Sciences software version 20.0 software (SPSS, Chicago, IL) was used for statistical analysis. The distribution of continuous variables to the normal distribution was evaluated using histogram and Q–Q plot test. Categorical variables are expressed as numbers and percentages and continuous variables are expressed as mean and standard deviation (SD) or median and interquartile range (IQR). The χ^2^ or the Fisher exact test was used to compare categorical variables. In the comparison of continuous independent variables, the Student *t* test was used for normally distributed variables and the Mann–Whitney *U* test was used for nonnormally distributed variables. A logistic regression model was created to identify risk factors for BSI in critically ill COVID-19 patients. Variables with a *P* value of <.20 in the univariate analysis and no moderate to high correlation with each other were included in the multivariate analysis. We included the following factors in the model: duration of COVID-19 disease, type of ICU, diabetes mellitus, APACHE-2 score, CVC, some laboratory values at the time of ICU admission (CRP, procalcitonin, d-dimer, and ferritin/procalcitonin rate). A *P* value <.05 was considered statistically significant.

## Results

In total, 195 patients were included in the study. The demographic and clinical characteristics of the patients are presented in (Table 1). In total, of 677 blood cultures were obtained from 195 patients. Blood cultures were obtained once from 82 patients (42.1%), 2 blood cultures from 31 (15.9%), 3 blood cultures from 24 patients (12.3%) and >3 blood cultures from 58 patients (29.7%). Culture positivity was detected in 173 (25.5%) of 677 blood cultures. Blood culture positivity was detected in 76 (39.0%) of 196 patients. Excluding blood-culture positivity considered as contamination, the prevalence of BSI in all 36 severe COVID-19 cases was 18.5% (Table 2).

**Table 1.** Demographics and Clinical Characteristics of the Patients

Characteristic	Total, No. (%)^a^
**Demographics**	
Age, mean ± SD	63.1±14.6
Sex, male	128 (65.6)
Comorbidities	
**Diabetes**	54 (27.7)
Hypertension	96 (49.2)
Cardiovascular disease	36 (18.5)
Chronic pulmonary disease	14 (7.2)
Chronic renal disease	25 (12.8)
Malignancy	28 (14.4)
At least 1 comorbid disease	145 (74.4)
**Clinical features at the time of admission**	
Fever ≥ 38°C	89 (45.6)
Cough	114 (58.5)
Shortness of breath	107 (54.9)
Sputum	20 (10.3)
Diarrhea	15 (7.7)
Symptom duration, median d (IQR)	5 (3–7)
**Clinical findings at the time of admission**	
Fever ≥ 38°C	51 (26.2)
Tachypnea >30	57 (29.2)
FiO2 (%), median (IQR)	27 (21–33)
**Laboratory values at the time of admission, median (IQR)**	
White blood cells, per mm^3^	5.960 (4,470–8,480)
Neutrophils, per mm^3^	4.140 (3,230–6,610)
Lymphocytes, per mm^3^	950 (730–1,270)
Platelets, × 10^3^/mm^3^, mean±SD	201.000±87.500
Creatinine, mg/dL	0.94 (0.74–1.34)
Ferritin, ng/mL	330 (172–601)
D-dimer, µg/mL	0.85 (0.49–1.74)
C-reactive protein, mg/L	78.4 (29.6–128.0)
Procalcitonin, ng/mL	0.14 (0.08–0.44)
**Antiviral treatment**	
Favipiravir	177 (90.8)
Favipiravir+ Hydroxychloroquine	5 (2.6)
Favipiravir+ Remdesivir	13 (6.7)
Antibacterial treatment	
Fluoroquinolone^b^	48 (24.6)
Macrolide^c^	6 (3.1)
Other^d^	109 (55.9)
No treatment	32 (16.4)

Note. SD, standard deviation; IQR, interquartile range; FiO_2_, fraction of inspired oxygen.

a Units unless otherwise stated.

b Levofloxacin and moxifloxacin are included.

c Clarithromycin and azithromycin are included.

d Cephalosporins, antipseudomonal β-lactam–β-lactamase inhibitors, carbapenems, glycopeptides, tetracycline, polymyxin group antibiotics are included.

**Table 2.** Evaluation of Blood Culture Positivity

Variable	Total, No. (%)
Patients with positive blood culture	76 (39)
Patients whose blood culture was evaluated as a contaminant	40 (20.5)
**Distribution of microorganisms considered as contaminants**	
Coagulase-negative *Staphylococcus*	34 (85.0)
*Corynebacterium* spp	5 (12.5)
*Bacillus* spp	1 (2.5)
Patients with bloodstream infections	36 (18.5)
**Distribution of bloodstream infection agents**	
Gram positive microorganisms	
Coagulase-negative *Staphylococcus*	12 (33.3)
Methicillin resistance	12
* Enterococcus spp.*	4 (11.1)
Vancomycin resistance	-
*Staphylococcus aureus*	3 (8.3)
Methicillin resistance	-
Gram negative microorganisms	
*Pseudomonas aeruginosa*	5 (13.9)
Carbapenem resistance	2 (5.6)
MDR	1 (2.8)
*Acinetobacter baumannii*	4 (11.1)
Carbapenem resistance	4 (11.1)
MDR	4 (11.1)
*Klebsiella pneumoniae*	4 (11.1)
Carbapenem resistance	3 (8.33)
MDR	3 (8.33)
*Escherichia coli*	1 (2.8)
Carbapenem resistance-MDR	-
*Enterobacter* spp	2 (5.6)
Carbapenem resistance-MDR	-
*Burkholderia cepacia*	1 (2.8)
MDR	1(2.8)

Note. MDR, Multi-drug resistance.

**Fig. 1.**
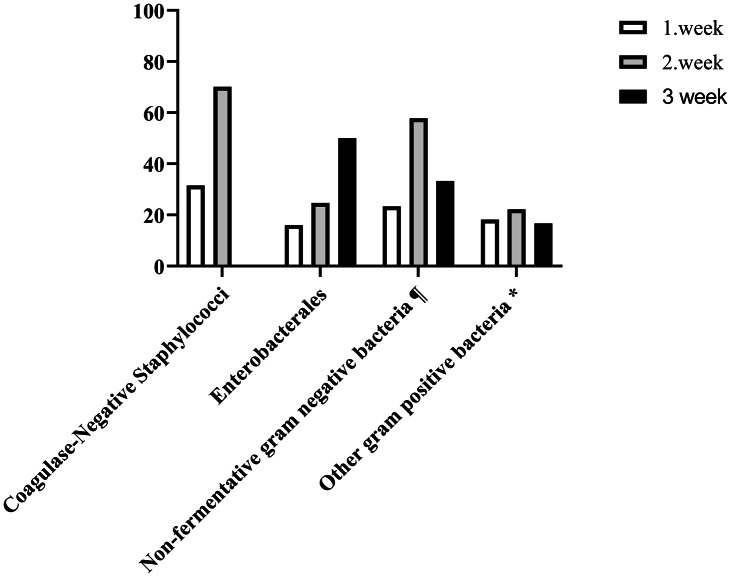
Distribution of microorganisms causing bloodstream infections according to the weeks of intensive care unit hospitalization. ¶ *Pseudomonas aeruginosa, Acinetobacter baumannii, Burkholderia cepacia.* *Staphylococcus aureus, Enterococcus spp.

In ICU patients, the prevalence of BSI was 30.6% (n = 26). Overall 21 (80.7 %) of these infections were considered primary BSIs and 5 infections (19.3%) were considered secondary BSIs. The source of secondary BSIs were respiratory system (3 patients), urinary system (1 patient), and soft-tissue infection (1 patient). 14 of the bloodstream infections are associated with gram positives and 12 with gram negatives. 83.3% (n = 10) of gram negative bacteremias were in multi-bed ICUs. The most frequently detected microorganisms in BSIs were coagulase-negative Staphylococci (n = 9, 34.6%), *A. baumannii* (n = 4, 15.4%), *Enterococcus* spp (n = 4, 15.4%), *K. pneumoniae* (n = 4, 15.4%), *P. aeruginosa* (n = 2; 7.7%), and other (n = 3, 11.4%), respectively (Fig. 1). The clinical and laboratory findings at the time of blood culture positivity of ICU patients are presented in Table 3.

**Table 3.** Clinical and Laboratory Characteristics of Critical COVID-19 Patients With BSI at the Time of Blood Culture Positivity

Variable	Total
Duration from the ICU admission to the occurrence of BSI, median d (IQR)	8.5 (3–13.5)
**Clinical findings, no. (%)**	
Fever	5 (19.2)
Vasopressor	12 (46.2)
SOFA score	9.1 ± 4.92
Invasive mechanical ventilation	17 (65.4)
Central venous catheter	14 (53.8)
Juguler	8 (57.1)
Femoral	4 (28.6)
Subclavian	2 (14.3)
**Laboratory parameters**	
White blood cells, per mm^3^ mean±SD	13.495 ± 6.882
Neutrophils, per mm^3^ mean±SD	12.245 ± 6.576
Lymphocytes, per mm^3^ mean±SD	706 ± 435
Platelets, ×10^3^/mm^3^, mean±SD	180.692 ± 89.400
C-reactive protein, mg/L, median (IQR)	102 (15–209)
Procalcitonin, ng/mL, median (IQR)	1.33 (0.23–4.84)
Lactate, median (IQR)	2.05 (1.72–2.82)
D-dimer, µg/mL, median (IQR)	3.23 (1.37–7.98)
Fibrinogen, mg/dL mean±SD	494 ± 177
Ferritin, ng/mL, median (IQR)	684 (512–1005)
Ferritin/Procalcitonin, median (IQR)	950 (345–4749)
IL-6, pg/mL, median (IQR)	66.6 (54.9–685)

Note. SD, standard deviation; IQR, interquartile range; BSI, bloodstream infection; ICU, ıntensive care unit; SOFA, Sequential Organ Failure Assessment score.

In multivariate analyses, CVC (OR, 8.17; 95% CI, 2.46–27.1; *P* < .01) and hospitalization in the multibed ICU (OR, 4.28; 95% CI, 1.28–14.3; *P* < .01) were risk factors associated with the acquisition of BSI (Table 4).

**Table 4.** Evaluation of Risk Factors for BSI in Critically İll COVID-19 Patients

Variable	BSI (+) (N=26)	BSI (−) (N=59)	*P* Value	Unadjusted OR (95% CI)	aOR (95% CI)
Age, mean ± SD	71.6 ± 11.8	65.3 ± 13.2	.038	1.04 (1.01–1.085)	N/A^a^
Sex, male, no. (%)	17 (65.4)	37 (62.7)	.814	1.123 (0.42–2.94)	…
**Comorbitidies**, no. (%)					
Diabetes	3 (11.5)	18 (30.5)	.062	3.36 (0.89–12.6)	0.310 (0.70–1.36)*
Hypertension	17 (65.4)	35 (59.3)	.597	0.77 (0.29–2.01)	…
Cardiovascular disease	7 (26.9)	16 (27.2)	.985	0.99 (0.35–2.80)	…
Chronic pulmonary disease	1 (3.8)	6 (10.2)	.431	2.83 (0.32–24.7)	…
Chronic renal disease	2 (7.7)	6 (10.2)	.535	1.35 (0.25–7.22)	…
Malignancy	3 (11.5)	5 (8.5)	.696	0.71 (0.15–3.22)	…
COVID-19–related symptom duration, median d (IQR)	10 (7.75–15.2)	9 (7–11.2)	.144	1.09 (0.99–1.20)	1.01 (0.88–1.15)*
Length of hospital stay, median d (IQR)^1^	3.5 (2–11.7)	3 (2–5)	.116	1.16 (1.03–1.31)	N/A^b^
**Clinical findings at the time of intensive care admission**					
APACHE-II, mean ± SD	16 ± 5.88	13.4 ± 5.98	.071	1.07 (0.99–1.15)	0.95 (0.84 –1.08)*
**Requirement of oxygen support, no. (%)**					
High flow nasal cannula	8 (30.8)	25 (42.4)	.708		…
Invasive mechanical ventilation	3 (11.5)	5 (8.5)		1.43 (0.40–5.05)	
Noninvasive mechanical ventilation	7 (26.9)	11 (18.6)		0.72 (0.22–2.27)	
Other	8 (30.8)	18 (30.5)		1.35 (0.25–7.07)	
Vasopressor	9 (34.6)	10 (16.9)	.072	0.38 (0.13–1.10)	N/A^c^
Central venous catheter	14 (53.8)	10 (16.9)	<.01	5.71(2.04–15.9)	8.17 (2.46 – 27.1)**
Acute kidney injury	17 (65.4)	18 (30.5)	.003	4.30 (1.61–11.4)	N/A^d^
Hemodialysis (CRRT)	7 (26.9)	7 (11.9)	.085	2.73 (0.84–8.83)	N/A^e^
Total parenteral nutrition	6 (23.1)	9 (15.3)	.383	1.66 (0.52–5.29)	…
Coinfection	10 (38.5)	11 (18.6)	.051	2.72 (0.97–7.61)	N/A^f^
Pneumonia	8 (30.8)	7 (11.9)	.035	3.30 (1.04–10.3)	3.16 (0.56 –17.5)*
**Laboratory parameters at the time of intensive care admission**					
Neutrophils, per mm^3^	9.945 (5.265–12.967)	7.780(4.740–11.520)	.227	1.07 (0.97–1.17)	…
Lymphocytes, per mm^3^	685 (387–955)	730(430–1.040)	.407	0.83 (0.33–2.10)	…
C-reactive protein, mg/L	113 (90–189)	101 (55–159)	.142	1.00 (0.99–1.01)	1.005 (0.99–1.01)
Procalcitonin, ng/mL	0.52 (0.15–1.28)	0.16 (0.08–0.46)	.005	1.01 (0.97–1.02)	0.98 (0.94–1.01)*
Ferritin, ng/mL	642 (288–1124)	506 (265–819)	.388	1.00 (0.99–1.00)	…
D-dimer, µg/mL	1.43 (0.96–3.5)	0.84 (0.54–1.60)	.06	1.15 (1.01–1.34)	1.10 (0.94–1.28)*
**Ferritin/procalcitonin**					
>800	14 (53.8)	47 (79.7)	.015	3.35 (1.23–9.10)	2.53 (0.70–9.04)*
≤800	12(46.2)	12 (20.3)			
**Anti-inflammatory treatments**					
Dexamethasone, n%	24 (92.3)	56 (94.9)	.639	0.643 (0.10–4.09)	…
Duration of dexamethasone treatment median, (IQR %25-75)	11.5 (9.75–18)	11 (9–15)	.660	1.01 (0.95–1.08)	…
Tocilizumab	10 (38.5)	21 (35.6)	.800	1.13 (0.43–2.93)	…
Tocilizumab and Dexamethasone	25 (96.2)	59 (100)	NA	…	…
**Type of ICU**					
Multibed room	20 (40.0)	30 (60.0)	.024	3.22 (1.13–9.16)	4.28 (1.28–14.3)**
Single rooms	6 (17.1)	29 (82.9)			
Hosmer-Lmeshow goodness of fit test: p = 0.889 –2 LogL: 82.3, Nagelkerke R^2^ : 0.326					

Note. SD, standard deviation; IQR, interquartile range; BSI, bloodstream infection; APACHE, Acute Physiology and Chronic Health Evaluation score; CRRT, continuous renal replacement therapy; ICU, ıntensive care unit.

a Age was not included the model due to the high correlation with APACHE II score (Pearson correlation coefficient: 0.505, p < 0.01).

b Duration of hospitalization was not included in the model due to the high correlation with duration of COVID-19 disease (Pearson correlation coefficient: 0.569, p < 0.01).

c Requirement of vasoactive agents was not included the model due to the high correlation with CVC (Pearson correlation coefficient: 0.730, p < 0.01).

d Acute kidney injury was not included in the model due to the high correlation with CVC (Pearson correlation coefficient: 0.590, p < 0.01).

e Hemodialysis was not included in the model due to the high correlation with acute kidney injury (Pearson correlation coefficient: 0.637, p < 0.01).

f Coinfection was not included in the model due to the high correlation with pneumonia (Pearson correlation coefficient: 0.808, p < 0.01).

1 Hospital stay before ICU admission in days.

*P ≥ .05; **P < .05.

Secondary bacterial (microbiologically confirmed) nosocomial infection was detected in 33 (38.8%) of critically ill COVID-19 patients. These are isolated BSIs (multi-bed ICU n = 12, single-bed ICU n = 0), BSI and bacterial pneumonia (multi-bed ICU n = 6, single-bed ICU n = 5) and bacterial pneumonia (multi-bed ICU n = 5, single-bed ICU n = 5), respectively. 77 (90.6%) of the critically ill COVID-19 patients received parenteral antibiotic therapy. Nosocomial bacterial infection incidence densities in COVID-19 single-room ICU, multi-bed ICU and non-COVID-19 intensive care units were 19.79, 45.99, and 23.4, respectively. In the COVID-19 and non-COVID-19 intensive care units, the patient/nurse ratio was 2/1. Hand hygiene compliance rates in COVID-19 single-room ICU, multi-bed ICU, and non-COVID-19 ICUs were 89.8%, 71.7%, and 94.7%, respectively.

## Discussion

In our study, the frequency of BSI was high in severe COVID-19 patients, especially in critically ill patients. BSI developed in one-third of critically ill patients. Although gram-positive microorganisms were more frequently detected, the frequency of gram-negative microorganisms increased after 1 week. Multibed ICU and the CVC were the most important risk factors for BSI.

The frequency of bacterial infection in COVID-19 patients is 6.9% on average, and this frequency can rise to 13.8% in ICU patients.^3^ In these patients, BSIs are the most common nosocomial infections after bacterial pneumonia.^3,5,6^ The frequency of BSI ranges between 1.6% and 7.9%.^8–10^ Compared to these data, the frequency of BSI was higher due to more homogeneous and critically ill patients in our study. In critically ill COVID-19 patients, the frequency of BSI up to 40.0% has been reported.^7,9,11,15^ Also, it is difficult to determine the frequency of BSI in these patients due to high blood-culture contamination.^8,16^ In our study, blood-culture positivity was considered as contamination in ∼20% of the patients. The stress of exposure to SARS-CoV-2 on healthcare workers and decreased compliance with standard infection control precautions and blood-culture procedures in contrast to respiratory isolation measures were thought to be associated with this high contamination rate.

BSI in COVID-19 patients often develops as a nosocomial infection, especially in ICU patients.^6,9,11^ Although the time of BSI in these patients varies between 1 and 37 days, BSIs generally develop after the first week of hospitalization.^9,11,17,18^ The risk of BSI increases depending on the length of stay in the ICU, and the cumulative risk rises above 50% in intensive care stays >30 days.^19^ In our study, BSIs developed as nosocomial infections within the first week after admission to ICU. Gram-positive microorganisms (eg, coagulase-negative staphylococci, *S. aureus*, and *Enterococcus* spp) are more frequently detected as BSI agents in COVID-19 patients.^6,8,10,16–18^ Similar to the literature data, gram-positive microorganisms were the most common BSI agents in our study. However, there was a relationship between the length of ICU stay and the distribution of BSI agents in our study. The frequency of gram-negative microorganisms in BSIs increased from the second week after admission to the ICU. This may be related to the increased frequency of patients colonization with gram-negative bacteria associated with long ICU hospitalization and low compliance with infection control measures. High nosocomial infection density and low hand hygiene compliance in COVID-19 ICUs, especially in multi-bed COVID-19 ICU, compared with non-COVID-19 ICUs support this hypothesis. Especially high nosocomial infection rates in multi-bed ICUs increase the inappropriate use of antibiotics. In our study, antibiotics were used in critically ill COVID-19 patients, although there was no proven nosocomial bacterial infection.

The follow factors are consdered risk factors for BSI in COVID-19 patients: disease severity, high oxygen support requirement, impaired mental status, intensive care support, length of stay in ICU, invasive mechanical ventilation, steroid and anticytokine therapy use, presence of CVC.^5–7,11,18,19^ Previous studies during the COVID-19 pandemic have shown that these factors increase the risk of developing nosocomial infections; increase in the burden on the health system; contribute to insufficient health workers; increase the number of health workers in intensive care with insufficient intensive care experience; and contribute to decrease in compliance with standard infection control measures.^6,20^ Patient follow-up in multibed ICUs increases the risk of nosocomial microorganism colonization and cross contamination as a result of decreased compliance with infection control measures.^21–23^ Interventions to improve adherence to vascular catheter insertion and care procedures, combined with standard infection control measures, have been shown to reduce hospital BSIs.^20,24,25^ In our study, the risk of BSI increased in patients with CVC, which is a risk factor for BSI, and in multibed ICUs where compliance with standard infection control measures decreased. We detected no correlation between steroid and anticytokines treatments and BSI.

Our study had several limitations. The first limitation is that the new ICUs where COVID-19 patients were followed during the pandemic eliminated the possibility of comparison with the pre–COVID-19 period. Also, compliance with standard infection control measures and invasive device insertion-care procedures, which was concluded to be the determining factor as a result of the study, could not be evaluated due to the retrospective character of the study. Third, the logistic regression model may not include all of the competing risk factors for BSIs. However, due to the retrospective nature of the study, a logistic regression model was created with available risk factors.

In conclusion, the prevalence of BSI in COVID-19 patients is particularly high in critically ill patients. Although BSIs frequently develop with gram-positive microorganisms, gram-negative microorganisms increased with the prolonged stay in an ICU. The CVC and multibed intensive care follow-up are risk factors for BSI. BSIs can be reduced by increasing compliance to infection control measures, and CVC insertion-care procedures. The use of single-bed ICUs where compliance can be achieved more effectively are important for the prevention of BSIs.
