# Evaluation of prevalence and risk factors for bloodstream infection in severe coronavirus disease 2019 (COVID-19) patients – CORRIGENDUM

**DOI:** 10.1017/ash.2022.272

**Published:** 2022-07-13

**Authors:** Kubra Erbay, Hasan Selcuk Ozger, Ozlem Guzel Tunccan, Ümmügülsüm Gaygısız, Merve Buyukkoruk, Fidan Sultanova, Mehmet Yıldız, Nazlıhan Boyacı Dündar, Müge Aydoğdu, Gulendam Bozdayi, Murat Dizbay

In the above article^1^, the original title:

“Evaluation of prevalance and risk factors for bloodstream infection in severe coronavirus disease 2019 (COVID-19) patients”

…contained a spelling error. The title has since been corrected to read:

“Evaluation of prevalence and risk factors for bloodstream infection in severe coronavirus disease 2019 (COVID-19) patients”

## References

[1] Erbay, K. , Ozger, H. , Guzel Tunccan, O. , Gaygısız, Ü , Buyukkoruk, M. , Sultanova, F. , … Dizbay, M. (2022). Evaluation of prevalence and risk factors for bloodstream infection in severe coronavirus disease 2019 (COVID-19) patients. Antimicrobial Stewardship & Healthcare Epidemiology, 2(1), E30. doi: 10.1017/ash.2021.254 PMC9614839 36310789

